# Accuracy of Intracranial Pressure Monitoring—Single Centre Observational Study and Literature Review

**DOI:** 10.3390/s23073397

**Published:** 2023-03-23

**Authors:** Adam I. Pelah, Agnieszka Zakrzewska, Leanne A. Calviello, Teodoro Forcht Dagi, Zofia Czosnyka, Marek Czosnyka

**Affiliations:** 1 Division of Neurosurgery, Department of Clinical Neurosciences, Addenbrooke’s Hospital, University of Cambridge, Cambridge CB2 0QQ, UK; 2 Neurosurgery, Mayo School of Medicine and Science, Rochester, MN 55905, USA; 3 School of Medicine, Dentistry & Biomedical Sciences, Queen’s University Belfast, Belfast BT7 1NN, UK; 4 Institute of Electronic Systems, Warsaw University of Technology, 00-65 Warszawa, Poland

**Keywords:** intracranial pressure, intraparenchymal sensor, zero drift, brain injury, ICP sensor, head trauma

## Abstract

Intracranial hypertension and adequacy of brain blood flow are primary concerns following traumatic brain injury. Intracranial pressure (ICP) monitoring is a critical diagnostic tool in neurocritical care. However, all ICP sensors, irrespective of design, are subject to systematic and random measurement inaccuracies that can affect patient care if overlooked or disregarded. The wide choice of sensors available to surgeons raises questions about performance and suitability for treatment. This observational study offers a critical review of the clinical and experimental assessment of ICP sensor accuracy and comments on the relationship between actual clinical performance, bench testing, and manufacturer specifications. Critically, on this basis, the study offers guidelines for the selection of ICP monitoring technologies, an important clinical decision. To complement this, a literature review on important ICP monitoring considerations was included. This study utilises illustrative clinical and laboratory material from 1200 TBI patients (collected from 1992 to 2019) to present several important points regarding the accuracy of in vivo implementation of contemporary ICP transducers. In addition, a thorough literature search was performed, with sources dating from 1960 to 2021. Sources considered to be relevant matched the keywords: “intraparenchymal ICP sensors”, “fiberoptic ICP sensors”, “piezoelectric strain gauge sensors”, “external ventricular drains”, “CSF reference pressure”, “ICP zero drift”, and “ICP measurement accuracy”. Based on single centre observations and the 76 sources reviewed in this paper, this material reports an overall anticipated measurement accuracy for intraparenchymal transducers of around ± 6.0 mm Hg with an average zero drift of <2.0 mm Hg. Precise ICP monitoring is a key tenet of neurocritical care, and accounting for zero drift is vital. Intraparenchymal piezoelectric strain gauge sensors are commonly implanted to monitor ICP. Laboratory bench testing results can differ from in vivo observations, revealing the shortcomings of current ICP sensors.

## 1. Introduction

A variety of intracranial pressure (ICP) sensors have been utilised to measure ICP and guide medical treatment. External ventricular drains (EVDs) are placed within the lateral ventricle to measure ICP pressure manometrically as a function of cerebrospinal fluid (CSF) pressure. They also serve to drain excess CSF to lower ICP and are often characterized as the “gold standard” of ICP measurement.

ICP measurement devices have also been designed around fiberoptic, piezoelectric strain gauge, and pneumatic microsensor technologies. Depending on the specific design, they can be inserted into the parenchyma, ventricle, or subarachnoid, subdural, or epidural spaces. While they cannot drain CSF, they are easier to implant and carry a lower risk of infection than EVDs [1]. It is worth noting that because of the inherent risks and costs associated with invasive ICP monitoring, there have been many attempts to replicate it non-invasively using methods such as transcranial doppler ultrasound (including machine learning) or correlation with tympanic membrane displacement, (amongst others) [2,3,4]. While there have been advancements in this subject, it is a different challenge and beyond the scope of this review.

The accuracy and precision of ICP measurement are critical factors in the use of ICP monitoring technologies. Accuracy can be difficult to ascertain both because of the physiology of ICP and because of the limitations of existing instrumentation [5]. Two exemplars of FDA-approved devices by the same manufacturer can yield different measurements when put in two different regions of the brain. Almost every ICP measurement device may drift. Some types may be re-zeroed in vivo, but not all. Why is this the case? Are such tolerances inevitable? What degree of reliability can be expected from existing ICP measuring instruments? This paper reviews the concepts and factors that bear on the accuracy of ICP measurements, including the reliability of ICP measuring technologies, and gives clinicians and researchers a view of the current state of monitoring and the choices available to them.

## 2. Materials and Methods

The observational study is based on findings made from TBI patients at the Neurosciences Critical Care Unit (NCCU) at Addenbrooke’s Hospital, Cambridge. From 1992 to 2019, 1200 patients were observed at the NCCU, providing insight into ICP monitoring and changes in the underlying technology. This is accompanied by a literature review on three key ICP monitoring considerations: zero drift, agreement amongst intraparenchymal sensors, and agreement between intraparenchymal sensors and CSF pressure. The review contains sources dating from 1960 to 2019. Sources considered to be relevant matched the keywords: “intraparenchymal ICP sensors”, “fiberoptic ICP sensors”, “piezoelectric strain gauge sensors”, “external ventricular drains”, “CSF reference pressure”, “ICP zero drift”, and “ICP measurement accuracy”.

## 3. Observational Study

### 3.1. Why Monitor ICP?

ICP measurement is utilised routinely in clinical practice when there is concern about pressure elevation or possibility of secondary injury and there are no contraindications [6,7,8,9,10,11]. Lundberg is widely credited with establishing the clinical paradigm for continuous ICP monitoring, with ventricular puncture, in the 1960s [12,13].

Historically, the study of ICP has been pursued most intensively in traumatic brain injury (TBI), but it is also commonly deployed in acute brain injury of vascular origin, such as subarachnoid haemorrhage and spontaneous intracerebral bleeding.

**Figure 1:**
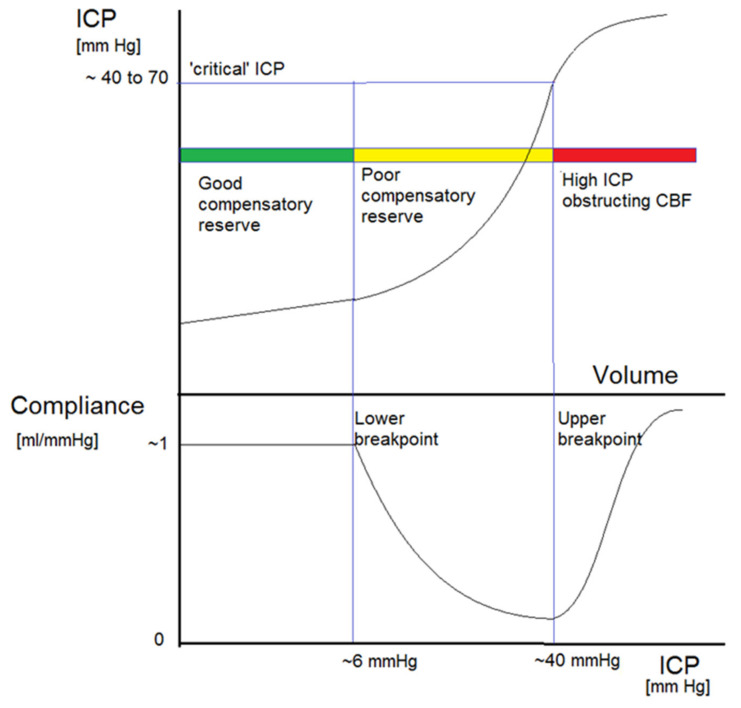
General shape of the pressure-volume curve (upper panel) and related brain compliance (change in intracranial volume over change in intracranial pressure, lower panel). There are three distinct zones. ICP first increases linearly with extra volume (zone of good compensatory reserve). Upon further volume load, the curve becomes exponential, indicating poor compensatory reserve. Past this zone, with further volume load, ICP is critically high, leading to arterial bed compression, decreased blood flow, and a high threat of brain ischaemia. This graph is a compilation of many previous works, starting from Lofgren and Zwetnow through Marmarou et al., and many more contemporary authors [21].

Head-injured patients often exhibit abnormal ICP dynamics. Elevated ICP interferes with cerebral blood flow (CBF), cerebral perfusion pressure (CPP), and cerebral compliance (Figure 1) [14,15,16]. Highly elevated ICP can result in cerebral ischaemia or herniation [14,17,18,19].

The Benchmark Evidence from South American Trials: Treatment of Intracranial Pressure (BEST:TRIP) trial, a multi-centre controlled trial with a cohort of 324 patients, found that treatment based on preventing ICP from rising above 20 mmHg was not superior to treatment based on imaging and examination. The authors maintain that ICP monitoring is critical to patient care [20].

The risks associated with ventriculostomy, which are shared by cervical and lumbar drains, include infection, CSF leak, interference from air bubbles, clots and debris, secondary injury, haemorrhage from improper insertion, and other complications of prolonged monitoring such as slit ventricle syndrome (for ventricular catheters) [22,23,24,25,26,27,28]. More modern intraparenchymal ICP sensors, typically implanted through a burr hole to a depth of about 2 cm, carry a lower risk of complications and correlate closely with intraventricular pressure [1,29,30].

The disadvantages of intraparenchymal monitoring are linked to the fact that they measure vectors of force within the parenchyma in the region surrounding the sensor rather than actual CSF pressure. These measurements are subject to distortion by several factors, including the direction of the vectors of force exerted on the sensor. Intraparenchymal ICP is not uniformly distributed within the intracranial compartment [31]. In theory, pressure measured in CSF follows Pascal’s law and is close to being equally distributed.

In non-communicating hydrocephalus, the pressure gradient between the ventricles and subarachnoid space has been reported to be on the order of 1–2 mm Hg or less [32].

**Figure 2:**
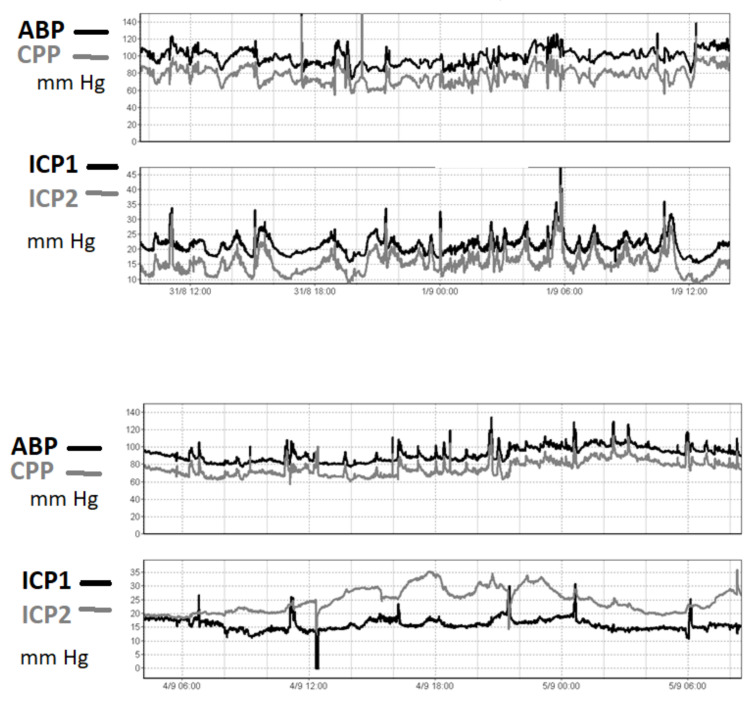
An ICP recording in one patient after TBI. ICP was recorded using two intraparenchymal microsensors (ICP- left hemisphere, ICP2- right hemisphere). In the upper panel, the two pressures are very well correlated in time, even though around 6 mm Hg of constant difference between the two readings is observed. In the lower panel, in contrast, the difference is seen to have increased to 20 mm Hg three days later. This patient suffered from diffuse brain injury without midline shift. The reason for the difference in readings was unknown. The true value of the ICP cannot be determined from these sensors.

Even though the correlation between two microsensors reporting simultaneously may vary over time and in terms of absolute value (Figure 2), intraparenchymal ICP measurements should be reliable.

**Figure 3:**
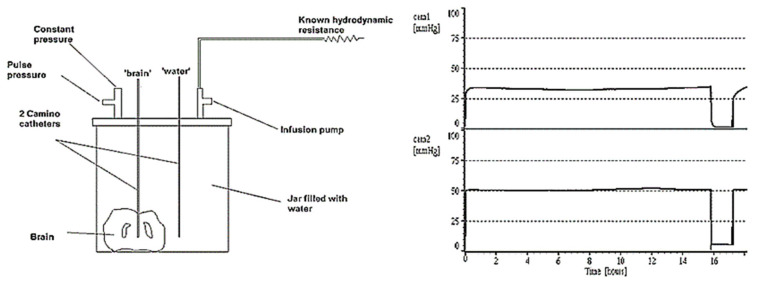
The ‘dead brain’ in a jar (pressurised externally). The microsensor in the brain tissue(cam1)shows a pressure measured at nearly 20 mm Hg lower than that of the water (cam2).

To illustrate how two ICP microsensors in the same brain may disagree, the following experiment was performed in the Cambridge laboratory. An animal brain was submerged in a sealed jar, and two microsensors were placed at the same depth beneath the top of the water column; one microsensor was inside the brain tissue and the other was in the surrounding water. When the jar was pressurised, the transducer in the water exhibited a pressure that was 20 mm Hg higher than in the brain. This constant difference was maintained over several hours (Figure 3). Pressure differences in the living brain may exist due to cerebral blood microcirculation, but at a much lower rate (microsensor tips are in a semi liquid extravascular environment).

### 3.2. Intracranial Pressure Sensor Technology

ICP can be monitored continuously by means of devices implanted in the ventricle, the parenchyma, the subdural and epidural spaces, the skull (but open to the subarachnoid space), the cisterna magna, or the lumbar subarachnoid space.

A pressure microtransducer, in contrast, is a device with an elastic or moveable component that deforms or moves when subjected to pressure and generates a signal. The signal is typically electrical and correlates with the pressure.

The three most common types of pressure transducers utilise piezoelectric, fiberoptic, and pneumatic sensing technologies. Piezoelectric sensors change their internal electrical resistance and produce electric signals when subjected to mechanical forces such as ICP [33]. Fiberoptic sensors incorporate a calibrated mirror that changes position in response to pressure. Reflected light is transmitted fiber-optically to a photoelectric device that generates electrical signals [29]. Pneumatic sensors typically consist of a small air-pouch balloon whose volume changes with pressure. These changes are translated into ICP measurements [33,34].

#### In Vivo Zeroing

Catheter systems can be calibrated or zeroed in vivo. Pressure transducers, in contrast, must be zeroed before implantation, with two exceptions: the Gaeltec™ epidural system (Gaeltec, Dunvegan, Isle of Skye, Scotland) and the Spiegelberg monitor (Spiegelberg, Hamburg, Germany), which was designed to allow in vivo zeroing [35].

**Figure 4:**
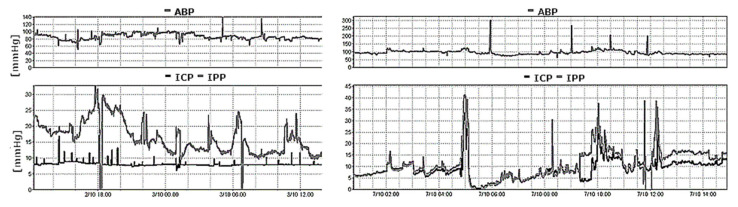
A recording of arterial blood pressure (ABP) and intraparenchymal pressure (IPP- bottom panel, grey line) together with EVD pressure (ICP- bottom panel, black line) using an external transducer in a patient after a poor-grade subarachnoid haemorrhage. The (**left** panel) demonstrates the results with the drain opened, whereas the right panel demonstrates the results with the drain closed. With an open EVD, the two pressure readings failed to correlate. EVD pressure is held constant at a value representing the calibrated level of the drain above the heart. With a closed EVD (**right** panel), the two measured pressure values correlate over time.

An EVD, when unclamped, is an open (to atmospheric pressure) system. In the event both an EVD and an intraparenchymal sensor are implanted at the same time in the same patient, the EVD must be closed for measurements to be comparable and to avoid environmental pressure interference, although there have been some recent attempts to develop catheters able to simultaneously measure ICP while draining CSF [36,37,38,39] (Figure 4).

### 3.3. Comparing ICP Sensor Performance to Bench Testing

**Figure 5:**
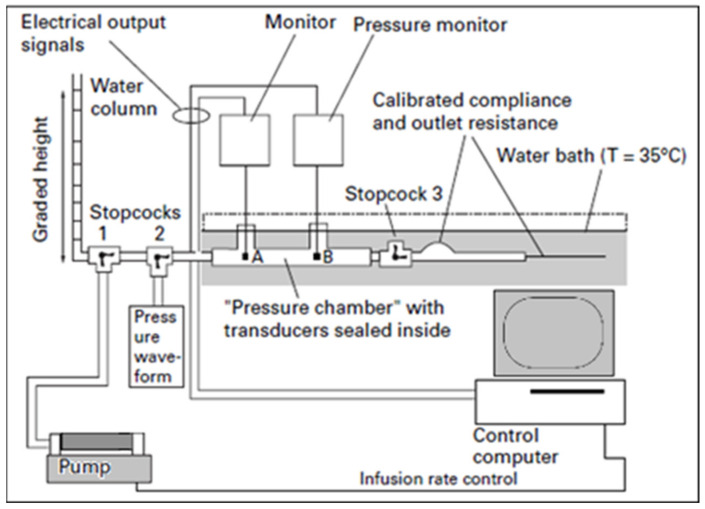
Bench Test Procedure: A bottle is filled with deionised water, leaving 20 mL of air to be removed during dynamic catheter testing. The bottle is then submerged horizontally in a water bath at a constant temperature of 35 °C. Static pressure on the bottle (representing pressure detected by ICP catheters) and reference static pressure (representing true ventricular pressure) are compared by changing the height of a water column in a 1.5 m graded vertical tube. Static pressure is released at intervals by allowing the water to flow out of an opened stopcock; conversely, pressure is increased by infusing fluid into the tubing [34,41].

Prior to regulatory approval, most ICP sensors undergo routine laboratory “bench testing” to confirm their performance relative to manufacturing specifications for zero drift standards and overall measurement accuracy. An example of this is the Cambridge experimental bench test procedure, described in Figure 5, which mimics CSF and physiological compliance [40].

Maximal zero drift measurements for a variety of ICP sensors were collected from the existing body of literature. They are summarised in Table A1 (see Appendix A). Table A2 and Table A3 display the literature-based comparisons of ICP sensors to each other and to CSF reference pressure, respectively (see Appendix A).

## 4. Literature Review

### 4.1. Zero Drift in ICP Sensors

The term “zero drift” refers to a category of drift in device calibration that can be remedied by resetting the zero point, which is usually not possible in situ, with exceptions. Error between true ICP and measured ICP of over 3 mmHg can potentially be critical, increasing in severity with error, and therefore zero drift is a significant consideration.

Every sensor is susceptible to zero drift. Large studies by [40,41,42,43] found varying drifts between a variety of sensor types. Refs. [40,41] reported <0.8 (mm Hg/day) zero drifts for several sensor types such as fiberoptic, piezoelectric, and pneumatic during a 24 h period. Refs. [42,43] found a larger range of 24 h drifts for these sensor types (2.1 mm Hg/day for pneumatic and 0.95 for fiberoptic), as well as testing extra-ventricular drains with reported drifts of 1.0–3.0 (HanniSet) and 2.0–4.0 (Medex) over a 10-day period. Other studies have found negligent zero drift amongst various sensor types ([44] reported <0.05 drift over 7 days for piezoelectric sensors). Please see Table A1 (Appendix A) for all zero drift studies reviewed.

### 4.2. Agreement between Intraparenchymal Sensors

Recent increases in the use of intraparenchymal sensors have brought into question how they compare to each other in a laboratory and in a clinical setting. Several studies have been conducted performing this comparison, most often between the Codman MicroSensor, Camino 110-4B, and Spiegelberg. Ref. [41] found that zero drift was not significantly different between the Codman MicroSensor and the Camino 110-4B, but the latter had a significantly higher temperature drift and higher static error (<2 mm Hg and <0.3 mm Hg, respectively). The study concluded that the Codman is preferred for clinical use. A second study by [40] found excellent agreement between the Codman MicroSensor and the Spiegelberg ICP monitor. Ref. [45] found a >10 mm Hg disparity between the Codman and Camino sensors in 10 patients but had a notably small sample size (*n* = 17), as did other studies. Please see Table A2 (Appendix A) for a full summary of the studies reviewed.

### 4.3. Agreement between Intraparenchymal Sensors and CSF Pressure in Clinical Studies

A series of studies have sought to ascertain the extent of agreement between popular intraparenchymal sensors and standard CSF pressure measurement. Several studies reported generally high agreement between intraparenchymal-measured ICP and CSF pressure. Ref. [46] reported R = 0.79 between Codman MicroSensor ICP and ventricular pressure in a study of 128 patients. Please see Table A3 (Appendix A) for a full summary of the studies reviewed.

## 5. Discussion

Intraparenchymal ICP probes, particularly the fiberoptic Camino 110-4B sensor, and strain gauge probes, particularly the Codman MicroSensor, are very popular amongst neurocritical care centers for TBI management. In a laboratory bench test [41], both the Camino and Codman sensors exhibited zero drift <0.8 mm Hg over 24 h at a static pressure of 20 mm Hg. In comparison, the Camino sensors were found to have significantly higher temperature drift than the Codman sensors [41].

In a paired comparison of clinical ICP recordings from the Camino and Codman sensors, however, the Codman was observed to be deviating by as much as 10 mm Hg in 18% of patients [45]. Another clinical assessment of the two sensors suggested >5 mm Hg differences in 13% of paired ICP recordings [47].

Paired measurements from the Codman MicroSensor and the Sophysa Pressio sensor have been reported to be in excellent agreement in a laboratory bench test setting, with a 7-day zero drift <0.05 mm Hg and static accuracy >0.5 mm Hg over the tested range of 0–100 mm Hg [34]. Clinical testing has yet to be completed.

A paired comparison of the Codman MicroSensor and the Raumedic Neurovent-P sensor revealed significant differences between baseline pressures (≥2 mm Hg in 96% of the Codman sensors and in 53% of the Raumedic sensors) due to either sudden or gradual shifts in baseline pressure. These measurement discrepancies were attributable to electrostatic discharges (0.5–5.0 kV) [48].

### 5.1. Overall Accuracy of ICP Sensors with Respect to CSF Reference Pressure

The efficacy of an ICP sensor for clinical use is dependent on its competence to accurately reflect ventricular CSF pressure. In one report, ICP readings from the fiberoptic Camino 110-4B sensor seemed to exceed true ventricular pressure by 1.15 mm Hg [49]. Another report indicated the mean differences to be as high as 9.2 ± 7.8 mm Hg [50].

Piezoelectric strain gauge sensors seem more accurate. Koskinen et al. [46] observed strong agreement between mean ventricular ICP and the Codman probe (18.3 ± 0.3 mm Hg vs. 19.0 ± 0.2 mm Hg, respectively) in a population of 128 neuro-critically ill patients. The Codman MicroSensor was also found to approximate lumbar CSF pressure in hydrocephalus patients, with measured differences of −0.75 ± 2.10 mm Hg [51].

The Spiegelberg pneumatic sensor exhibited an absolute difference of 3 mm Hg between the transducer and intraventricular pressure. The Spiegelberg sensor was also reported to produce ICP values 10% lower than the reference pressure, especially when ICP was greater than 25 mm Hg [52].

### 5.2. Future of ICP Sensors

In the future, ICP monitoring devices may be internet-connected or telemetric and possibly non-invasive. This is a large area of research already, as clinicians and researchers aim to reduce risk and allow more patients to be monitored. In vivo zeroing as standard is an additional goal, with some companies already releasing monitors capable of doing so, as previously mentioned.

## 6. Conclusions

Precise ICP monitoring is a key tenet of neurocritical care. Intraparenchymal piezoelectric strain gauge sensors are commonly implanted to monitor ICP. However, the measured intraparenchymal pressure is not always equal to the ‘real’ ICP (pressure measured in CSF). The average discrepancy may be +/− 6 mm Hg. Accounting for zero drift is vital but not trivial. Laboratory bench testing reveals the shortcomings of current ICP sensors, although the results from bench tests may not always compare to in vivo observations. Selection of an ICP monitor is an important and significant decision to make, and one that is not always clear due to the many differences between sensors’ ease of use, accuracy, and invasiveness. Therefore, it is important to continually revisit the performance of ICP monitors to optimise sensor and monitoring recommendations as ICP monitoring technology evolves [53,54,55,56,57].

## Data Availability

The data presented in this study are available on request from the corresponding author. The data are not publicly available due to privacy protection.

## Glossary

ICP	Intracranial Pressure
TBI	Traumatic Brain Injury
EVD	External Ventricular Drain
CSF	Cerebrospinal Fluid
CPP	Cerebral Perfusion Pressure

## Appendix A

Table A1 highlights differences in zero drift in measurement found between popular intraparenchymal ICP sensors and extra-ventricular drains in studies of TBI patients; these results may influence the decision to introduce one sensor over another in clinical practice. Each sensor is susceptible to zero drift, with comparative observations presented here. mm Hg—millimeters of mercury.

**Table A1:** Comparison of Zero Drift Among Different ICP Sensors. mm Hg—millimeters of mercury.

Laboratory	Reference	Sensor	Sensor Type	Maximal Drift (mm Hg/day)	Comments
Monza, Italy	Citerio et al. (2004) [58]	Raumedic Neurovent-P	Piezoelectric Strain Gauge	0–2.0	Overall drift past 5 days; precise measurements for long-term, continuous recording
	Citerio et al. (2008) [59]	Raumedic Neurovent-P	Piezoelectric Strain Gauge	±3.0	Clinical application of Citerio et al. (2004) [50]; 12–17% failure of sensor to accurately measure ICP (*n* = 99)
Cambridge, UK	Czosnyka et al. (1996) [41]	Camino 110-4B	Fiberoptic	<0.8	24 h period
	Czosnyka et al. (1996) [41]	Codman MicroSensor	Piezoelectric Strain Gauge	<0.8	24 h period
	Czosnyka et al. (1996) [41]	InnerSpace Medical ICP Monitoring Catheter Kit (OPD-SX)	Spectral Frequency	<0.8	24 h period; zero drift <0.4 mm Hg measured at a static pressure of 0 mm Hg
	Czosnyka et al. (1997) [40]	Camino 110-4B	Fiberoptic	<0.7	24 h period
	Czosnyka et al. (1997) [40]	Spiegelberg	Pneumatic	<0.7	24 h period; hourly adjustments to zero produced <0.3 mm Hg drift
	Allin et al. (2008) [34]	Sophysa Pressio	Piezoelectric Strain Gauge	<0.05	Over a 7-day period
	Allin et al. (2008) [34]	Codman MicroSensor	Piezoelectric Strain Gauge	<0.05	Over a 7-day period
	Al-Tamimi et al. (2009) [44]	Codman MicroSensor	Piezoelectric Strain Gauge	2.0	Median value; 108 in-situ hours (median); drift was found to increase over time (Spearman’s correlation coefficient = 0.342; *p* = 0.001); drift ≥ 5.0 mm Hg found in 20% of sensors
Santiago de Compostela, Spain	Gelabert-González et al. (2006) [60]	Camino 110-4B	Fiberoptic	7.3 ± 5.1	Mean value; clinical assessment of 1000 sensors: 79 sensors (12.6%) showed no zero drift on removal; mean monitoring time of 58.4 ± 8.6 h
Southampton, UK	Gray et al. (1996) [53]	Codman MicroSensor	Piezoelectric Strain Gauge	0–1.0	24 h period; sensors inserted in both parenchymal (mean zero drift: 0.312 mm Hg) and subdural (mean zero drift: 0.475 mm Hg) locations
Umeå, Sweden	Koskinen et al. (2005) [46]	Codman MicroSensor	Piezoelectric Strain Gauge	0.9 ± 0.2	Zero drift not correlated with duration of monitoring (analysis of data recorded over 7.2 ± 0.4 days; *p* = 0.9, Pearson *R* = 0.002)
Frankfurt am Main, Germany	Lang et al. (2003) [52]	Spiegelberg	Pneumatic	≥±2.0	Average monitoring time of 10 days; sensors inserted in both intraparenchymal and subdural locations
Barcelona, Spain	Martínez-Mañas et al. (2000) [55]	Camino 110-4B	Fiberoptic	0 ± 2.0 in the first 24 h, then <±1.0 per day	56 probes tested to confirm manufacturer specifications; 60.71% complied with zero drift standards, 39.28% drifted to positive or negative values; no observed correlation between monitoring duration and zero drift (*p* = 0.27)
Teubingen, Germany	Morgalla et al. (1999) [42]	Camino 110-4B	Fiberoptic	1.0–2.0	Microsensor accuracy was reported: 24 h period (0.80 mm Hg drift), measurements binned at 5 mm Hg pressure intervals in the range of 0–80 mm Hg; 10-day drift measured at the same intervals (8.0 mm Hg)
	Morgalla et al. (1999) [42]	Codman MicroSensor	Piezoelectric Strain Gauge	4.0≥	Microsensor accuracy was reported: discrepancies observed at pressures ≥60 mm Hg; 24 h period (0.95 mm Hg drift), measurements binned at 5 mm Hg pressure intervals in the range of 0–80 mm Hg; 10-day drift measured at the same intervals (2.0 mm Hg)
	Morgalla et al. (1999) [42]	Epidyn	Epidural	>8.0	Microsensor accuracy was reported: underestimated ICP, especially at higher pressures; 24 h period (1.20 mm Hg drift), measurements binned at 5 mm Hg pressure intervals in the range of 0–80 mm Hg; 10-day drift measured at the same intervals (15.0 mm Hg)
	Morgalla et al. (1999) [42]	Gaeltec ICT/B	Epidural	4.0≥	Microsensor accuracy was reported: 24 h period (1.5 mm Hg drift), measurements binned at 5 mm Hg pressure intervals in the range of 0–80 mm Hg; 10-day drift measured at the same intervals (10 mm Hg)
	Morgalla et al. (1999) [42]	HanniSet	External Ventricular Drain	1.0–3.0	Microsensor accuracy was reported: 24 h period (0.2 mm Hg drift), measurements binned at 5 mm Hg pressure intervals in the range of 0–80 mm Hg; 10-day drift measured at the same intervals (1.0 mm Hg)
	Morgalla et al. (1999) [42]	Medex	External Ventricular Drain	2.0–4.0	Microsensor accuracy was reported: 24 h period (1.8 mm Hg drift), measurements binned at 5 mm Hg pressure intervals in the range of 0–80 mm Hg; 10-day drift measured at the same intervals (3.5 mm Hg)
	Morgalla et al. (1999) [42]	Spiegelberg	Pnematic	<4.0 at pressures >50mm Hg; ≤6.0 at pressures >60 mm Hg	Microsensor accuracy was reported: 24 h period (2.1 mm Hg drift), measurements binned at 5 mm Hg pressure intervals in the range of 0–80 mm Hg; 10-day drift measured at the same intervals (7.0 mm Hg)
	Morgalla et al. (2002) [38]	Camino 110-4B	Fiberoptic	2.9	Median values for mean absolute pressure changes; 10-day drift: 4.0 mm Hg (transducers tested at 0–50 mm Hg)
	Morgalla et al. (2002) [43]	Gaeltec ICT/B	Epidural	5.2	Median values for mean absolute pressure changes; 10-day drift: 9.0 mm Hg (transducers tested at 0–50 mm Hg)
	Morgalla et al. (2002) [43]	HanniSet	External Ventricular Drain	0	Median values for mean absolute pressure changes; 10-day drift: 0 mm Hg (transducers tested at 0–50 mm Hg)
	Morgalla et al. (2002) [43]	Spiegelberg	Pneumatic	2.4	Median values for mean absolute pressure changes; 10-day drift: 2.0 mm Hg (transducers tested at 0–50 mm Hg)
Copenhagen, Denmark	Norager et al. (2018) [27]	Raumedic Neurovent-P	Piezoelectric Strain Gauge	2.5	Median baseline drift in 19 sensors (median implantation time of 241 days)
	Lilja et al. (2014) [54]	Raumedic Neurovent-P	Piezoelectric Strain Gauge	±2.0	Assessment of hydrocephalus patients (*n* = 21); median duration of sensor implantation was 288 days; poor compatible ICP curve visualization software
Glasgow, UK	Piper et al. (2001) [56]	Camino 110-4B	Fiberoptic	−0.67	Mean zero drift (3-day median implantation time); median drift reported at −1 mm Hg; more than 50% of the catheters had an observed drift >±3 mm Hg

Table A2 highlights the main differences in measurement capacity found between popular intraparenchymal ICP sensors in laboratory* and clinical** studies of TBI patients; these results may influence the decision to introduce one sensor over another in clinical practice.

**Table A2:** Agreement between Intraparenchymal ICP Sensors. ICP—intracranial pressure, mm Hg—millimeters of mercury.

Laboratory	Reference	Sensor 1	Sensor 2	Agreement	Comments
Cambridge, UK	Allin et al. (2008) [34]	Codman MicroSensor	Sophysa Pressio	Excellent agreement (reported Pearson *R* = 0.999)	Codman devices require additional bridge amplifiers to connect to computerized data streaming
	Czosnyka et al. (1996) [41]	Camino 110-4B	Codman MicroSensor	No significant differences in zero drift at a static pressure of 20 mm Hg; comparable for pulsatile pressure measurement; Camino temperature drift (0.27 mm Hg/°C) significantly higher than Codman; <0.3 mm Hg static error (Camino) vs. <2 mm Hg static error (Codman); very good frequency detection for both (bandwidth >30 Hz)	Codman is preferred for clinical use; also bench tested InnerSpace Medical’s ICP Monitoring Catheter Kit (OPX-SD), which had the lowest 24 h zero drift compared with both Codman and Camino sensors, but otherwise did not perform as well
	Czosnyka et al. (1997) [40]	Camino 110-4B	Spiegelberg	Camino temperature drift recorded at 0.27 mm Hg/°C; excellent agreement between transducers at pressures 0–100 mm Hg over 20 min (reported Pearson *R* = 0.99); static error <1 mm Hg up to pressures of 40 mm Hg that increased to 5 mm Hg at 100 mm Hg (Spiegelberg) vs. static error <0.7 mm Hg (Camino)	Spiegelberg devices are less expensive but are “limited by low frequency response and non-linear distortion as amplitude underestimation increases [with] mean pressure”
Newcastle upon Tyne, UK	Banister et al. (2000) [45]	Camino 110-4B	Codman MicroSensor	ICP measured within 10 mm Hg in 11 patients; >10 mm Hg disparity in 6 patients	Small sample size (*n* = 17); Codman was “misleading” in 18% of patients; preference for Camino sensors to register clinical events
Oslo, Norway	Eide (2006) [47]	Camino 110-4B	Codman MicroSensor	Differences >5 mm Hg observed in 13% of ICP recordings	Extremely small sample size (*n* = 3); discrepancies attributed to differing baseline pressures
	Eide & Bakken (2011) [48]	Codman MicroSensor	Raumedic Neurovent-P	Differences in baseline pressure ≥2 mm Hg in 96% of Codman sensors and 53% of Raumedic sensors observed as a result of electrostatic discharges (0.5–5 kV)	Discrepancies in baseline pressures (either sudden or gradual shifts) ≥10 mm Hg can significantly affect ICP management

Table A3 highlights the agreement found between intracranial pressure measured by popular intraparenchymal ICP sensors and CSF pressure in studies of TBI patients; these results may influence the decision to introduce one sensor over another in clinical practice.

**Table A3:** Agreement between Intraparenchymal Sensors and CSF Pressure in Clinical Studies. CSF—cerebrospinal fluid, ICP—intracranial pressure, and mm Hg—millimeters of mercury.

Laboratory	Reference	Sensor	Differences from CSF Pressure	Comments
Oslo, Norway	Brean et al. (2006) [61]	Codman MicroSensor	Mean difference between Codman and ventricular reference pressure reported at −0.71 ± 6.8 mm Hg	Data obtained from a case study; measurements from single wave parameters
	Eide et al. (2012) [62]	Codman MicroSensor, Edward’s fluid sensor connected to an external ventricular drain (Truwave PX-600 F Pressure Monitoring Set, Edwards Lifesciences LLC, Irvine, CA, USA), and Spiegelberg	Significant differences in mean ICP reported >5 mm Hg between ventricular pressure and each sensor type	Comparison of solid strain gauge sensors with either fluid or air-pouch sensors; “simultaneous monitoring of ICP using two solid sensors may show marked differences in static ICP but close to identity in dynamic ICP waveforms”; solid ICP sensors exhibit less disparity from “true” ICP and are preferred for clinical use; small sample size (*n* = 17)
Marseille, France	Bruder et al. (1995) [63]	Camino 110-4B	Camino underestimated ventricular pressure by about 9 mm Hg	95% confidence interval of bias: −9.8 to 27.8 mm Hg; small sample size (*n* = 10), male patients only
Newcastle upon Tyne, UK	Chambers et al. (1993) [49]	Camino 110-4B	Reads an average of 1.15 mm Hg higher than ventricular pressure	
	Chambers et al. (2001) [64]	Spiegelberg	Mean ICP differences > ±1.5 mm Hg between Spiegelberg and ventricular pressure	Reported results obtained from 10 patients; small overall sample size (*n* = 11)
Sheffield, UK; Singapore	Childs & Shen (2015) [65]	Raumedic Neurovent-P	Mean difference between intraparenchymal and ventricular pressure measured at −0.832 mm Hg	Tissue pressure is reported to be marginally lower than ventricular pressure (*p* = 0.379); temperature also did not vary significantly between local pressure sites (*p* = 0.92); small sample size (*n* = 17)
Houston, TX, USA	Crutchfield et al. (1990) [66]	Camino Model 420	Camino estimated ventricular pressure within ±3 mm Hg over a 0- to 30-mm Hg pressure range; robust correlation of 0.977	Study conducted in dogs
	Gopinath et al. (1995) [67]	Codman MicroSensor	Mean difference between Codman and ventricular pressure measured at 0.5 ± 2.6 mm Hg	Small sample size (*n* = 25)
Frankfurt am Main, Germany	Lang et al. (2003) [52]	Spiegelberg	Absolute difference between Spiegelberg and intraventricular pressure >±3 mm Hg in 99.6% of paired readings and >±2 mm Hg in 91.3% of paired readings	Average Bland Altman bias of 0.5, with 10% lower Spiegelberg readings with ICP > 25 mm Hg (*n* = 87)
Umeå, Sweden	Koskinen et al. (2005) [46]	Codman MicroSensor	Strong agreement between the Codman and ventricular pressure (*p* < 0.0001, Pearson *R* = 0.79)	Mean ICP in the ventricles measured at 18.3 ± 0.3 mm Hg vs. 19.0 ± 0.2 mm Hg measured by Codman (*n* = 128)
	Lenfeldt et al. (2007) [51]	Codman MicroSensor	Measured differences between Codman and lumbar pressure observed at −0.75 ± 2.10 mm Hg	Agreement between intracranial and lumbar pressure assessed patients with normal pressure hydrocephalus (*n* = 10)
Orange, CA, USA	Schickner & Young (1992) [50]	Camino 110-4B	Mean ICP difference between the Camino and the ventricular catheter of 9.2 ± 7.8 mm Hg	ICP recorded for up to 118 h; small sample size (*n* = 10)
